# Lacrimal gland pleomorphic adenoma: a narrative review

**DOI:** 10.5935/0004-2749.2022-0057

**Published:** 2022-10-19

**Authors:** Lucas Horochoski, Guilherme Warmling Schulz, Andrei Koerbel

**Affiliations:** 1 Faculdade de Medicina, Universidade da Região de Joinville, Joinville, SC, Brazil; 2 Departamento de Neurocirurgia, Instituto de Neurociência, Joinville, SC, Brazil

**Keywords:** Adenoma, pleomorphic, Lacrimal apparatus, Salivary gland neoplasms, Orbit, Biopsy, fine-needle, Adenoma pleomorfo, Aparelho lacrimal, Neoplasias da glândulas salivares, Órbita, Biopsia por agulha fina

## Abstract

We present a literature review of 57 publications describing this pathology, published from the year 2012. In all these studies patients were reported to depict a slow-growing, motionless mass, which is painless at most times. All cases were managed by total excision, except for one report where adjuvant radiotherapy was applied. Among the several therapeutic strategies, the total tumor resection, preserving the tumor pseudocapsule intact, appears to be a consensus in treating the disease efficiently. Furthermore, fine-needle aspiration biopsy, including the assessment of genetic alterations, has proved to be a valuable tool in the diagnosis of challenging cases. Our literature survey also suggests that an incisional biopsy before the surgery may lead to the pseudocapsule disruption, thus considerably increasing the chances of adenoma recurrence, enabling its malignization. At present, genetics studies indicate that the molecular aberrations involved in the adenoma are similar to those represented in the salivary gland tumor pathogenesis. Further, in the recurrent cases, the pathology becomes difficult to treat and multiple surgeries may be required, occasionally, leading to radical surgery treatment.

## Abstract (pt)

Uma revisão narrativa da literatura de 57 publicações que descrevem esta patologia, publicada a partir de 2012. Os pacientes têm uma massa de crescimento lento e imóvel, que na maioria das vezes é indolor. Todos os casos foram tratados por excisão total, com exceção de um relatório de radioterapia adjuvante. Entre as estratégias terapêuticas encontradas, a ressecção total do tumor, preservando a pseudocápsula tumoral intacta, parece ser um consenso. Alternativamente, a biópsia por aspiração de agulha fina incluindo a avaliação de alterações genéticas pode representar uma ferramenta valiosa nos casos diagnósticos desafiadores. Uma biópsia incisional antes da cirurgia não é recomendada, pois a ruptura da pseudocápsula aumenta consideravelmente a recorrência do adenoma, permitindo até mesmo sua malignização. Com relação à genética, estudos atuais indicam que as aberrações moleculares envolvidas no adenoma são semelhantes às da patogênese do tumor da glândula salivar. Para casos de recorrência, a patologia torna-se difícil de tratar e múltiplas cirurgias podem ser necessárias, às vezes levando a um tratamento cirúrgico radical.

## INTRODUCTION

Lacrimal gland pleomorphic adenoma (LGPA) is a disease that affects the human orbital region. It is a type of benign tumor composed of epithelial and myoepithelial elements, with considerable variations in the appearance and proportions of these components^(1,2,3,4^, ^5,6,7)^. Most incidences of this disease occur within the lacrimal gland, 84%-90% occur in the orbital lobe and the remaining cases in the palpebral lobe^(3,5,8,9,10,11,12^, ^13)^. Few very rare cases have been reported to occur in other locations that contain accessory or ectopic lacrimal gland tissue, such as those occurring in the eyebrow, eyelids away from the eyelid lobe, and intraocularly^(3,8,14,15)^. LGPA constitutes most benign lacrimal gland epithelial tumors, and it represents the greater part of all lacrimal gland epithelial tumors^(1,2^, ^3,5,8,9,11,13,16,17)^. This tumor is known to affect patients with an average age of 40 years. However, the disease range can vary starting from early childhood to the 90s. Moreover, there is no particular evidence of greater predisposition to this disease according to race or geographic location^(3,13)^.

Patients with LGPA typically present symptoms of a slow-growing, painless orbital mass, occasionally acute orbital inflammation as well, with nonaxial proptosis, diplopia, mechanical ptosis, and reduced vision. The average duration of symptoms is approximately two years^(1^, ^2,3,6,10,13,18)^, and pain as well as inflammation are uncommon. Contrast computed tomography (CT) in patients with LGPA generally shows a well-defined, solid oval, or round mass. Remodeling of the adjacent bone has been suggested, with an expansion of the lacrimal fossa, an occasional calcification, and cystic change^(12,13)^. The internal architecture often appears homogeneous on CT and heterogeneous on the magnetic resonance image^(1,3,6,13)^. However, the differential diagnosis in the case of other intraorbital lesions can be difficult since they are specific to the lacrimal gland or originate in adjacent tissues. Some examples are vascular tumors (e.g., hemangioma), rhabdomyosarcomas, lymphoid tumors, dermoid and epidermoid cysts, and metastases^(19,20)^.

This study aims to determine the intricacies of LGPA pathology and analyze the state-of-the-art literature evidence to identify the characteristics of LGPA and the patterns associated with it. Moreover, it aims to disseminate the knowledge necessary to make the differential diagnosis of LGPA more efficient. This study supports seeking the best therapeutic option for patients with LGPA enabling the best possible progress, minimal morbidity, and less risk of recurrence.

## METHODS

The present study was initiated by searching for articles related to the theme on virtual platforms, such as ScienceDirect, PubMed, Scielo, EBSCO, and LILACS. For this purpose, the set of terms “pleomorphic adenoma” + “lacrimal gland” was used. The results were filtered for articles published since 2012 (10 years) for review articles, research articles, case reports, and mini-reviews. 132, 33, 5, 28, and 4 were found, respectively, on each of the platforms described above.

On the basis of the abovementioned search criteria and our primary analysis, we further excluded duplicate papers and articles, which indicated a conflict of interest. Moreover, the papers written in languages other than English, Spanish, or Portuguese were eliminated from the study, along with those that were not directly related to the subject of this study. We complemented our electronic search with three book chapters; two related to the orbit anatomy and surgery approaches and one related to classifications of tumors of the eye acquired, particularly from the World Health Organization (WHO). The literature review for this study included 56 publications in total (24 case reports, 12 original contributions, 9 reviews, 5 clinical research, 3 book chapters, 1 thesis, 1 experimental study, and 1 retrospective case series).

## RESULTS

The literature review revealed that the primary LGPA complaint is the presence of a mass growth in the superolateral region of orbit, typically causing globe inferomedial displacing, and compromising vision acuity. Lacrimal gland lesions are considered relatively uncommon^(18)^, and as reported by Von Holstein et al., these lesions appear at an average annual incidence rate of approximately 1.3 per 1,000,000 people in Denmark^(18)^. Furthermore, the statistics state that the benign neoplasms represent 22.8% of the cases, and more specifically the LGPA account for 13.4% of the cases, with a calculated incidence rate of 1.74/10,000,000 per year^(18)^.

**Figure 1:**
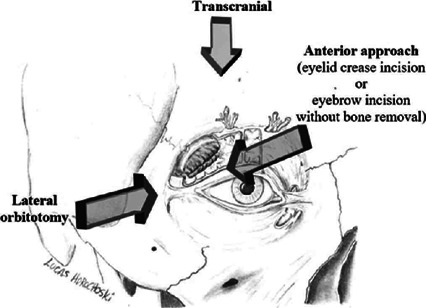
Schematic drawing, by the authors, showing the entrance points of the main approaches to lacrimal gland pleomorphic adenoma (LGPA).

The LGPA is known to occur typically only on one side, with no apparent left or right predominance described. Although, in our study, the literature gathered indicated more occurrence of the right lacrimal gland LGPA (left [n=10], right [n=14]). All LGPA cases resolved through total excision, except for one which required the support of adjuvant radiotherapy, indicative of a malignant component^(21)^. There were six case reports of preoperative incisional biopsy^(16,22,23,24,25^, ^26)^. The surgical approaches were varied (Figure 1), and the most frequently used was the lateral orbitotomy. The other methods were the anterior orbitotomy, or the transcranial approach^(8,10,11,16,19,20,21,22^, ^23,24,25,26,27^, ^28,29,30,31,32^, ^33,34,35,36,37)^.

According to prior reports incidence of LGPA is slightly predominant in men (n=14) compared in women (n=10)^(10,11,22)^. However, few studies have indicated that it shows an equal distribution between men and women^(13,19,29)^, with a mean age at diagnosis of 44.0 ± 23.5 (mean ± SD) years old, ranging from 7^(23)^ to 81^(25)^. Reports also demonstrated a lower rate of incidence in pediatric patients^(10,11,19,20,23)^. A summary of these findings has been shown in table 1. To date, there are only two reported cases of tumor necrosis^(21,29)^ and a single case report of ectopic LGPA^(8)^; however, 24 different cases were previously described by Mulay et al.^(15)^ in their study conducted on accessory lacrimal gland tumors.

**Table 1:** Cases report included in our analysis of LGPA

Study	Sex	Age	Side	Necrosis	Pain	Visual loss	Biopsy	Therapeutic strategy	Surgical approach
Adekunle et al.^(25)^	F	81	R			-	+	TR	Anterior orbitotomy
Alam et al.^(16)^	M	34	L	-	-	+	+	TR	Anterior orbitotomy (Eyelid crease incision)
Alsuhaibani et al.^(8)^	F	75	L	-	-	+	-	TR	Anterior orbitotomy (Eyelid crease incision)
Ayala et al.^(19)^	F	13	L	-	-	-	-	TR	Lateral orbitotomy
Binatli et al.^(27)^	M	62	R		-	-	-	TR	Transcranial
Bryant et al.^(28)^	M	16	R		-	-	-	TR	Transcranial
Casado et al.^(29)^	M	48	R	+	+	-	-	TR	Anterior orbitotomy
Chen et al.^(30)^	M	40	R			+	-	TR	Lateral orbitotomy
Guerra et al.^(22)^	M	24	L	-	-	+	+	TR	Anterior orbitotomy
Gupta et al.^(23)^	M	7	L		-	-	FNAB	TR	Lateral orbitotomy
Iyeyasu et al.^(20)^	F	73	L	-		+	-	TR	Lateral orbitotomy
Jakobiec et al.^(31)^	F	49	R		-	-	-	TR	Eyelid crease incision
Korchak et al.^(24)^	M	9	L			-	+	TR	Lateral orbitotomy
Misra et al.^(32)^	M	62	R		-	+	-	TR	Lateral orbitotomy
Moraru et al.^(33)^	M	51	R		-	-	-	TR	Lateral orbitotomy
Pakdel et al.^(10)^	M	68	R	-	+	+	-	TR	Lateral orbitotomy (modified Stallard incision)
Pokharel et al.^(26)^	M	15	R		+	+	FNAB	TR	Lateral orbitotomy
Porto et al.^(11)^	M	68	R	-	+	-	-	TR	Anterior orbitotomy
Rinna et al.^(34)^ Case 1	F	50	L		-	+	-	TR	Lateral orbitotomy
Rinna et al.^(34)^ Case 2	F	44	L		-	-	-	TR	Anterior orbitotomy (Eyelid crease incision)
Skippen et al.^(35)^	F	54	R		-	-	-	TR	Lateral orbitotomy
Sung et al.^(21)^	M	69	L	+	-	-	-	TR + RT	Lateral orbitotomy
Vijayakumar^(36)^	F	11	R		-	-	-	TR	Lateral orbitotomy
Wajda et al.^(37)^	F	35	R			-	-	TR	Anterior orbitotomy

Subtitle: M= Male; F= Female; L= Left; R= Right; TR= Tumor Resection; RT= Radiotherapy; FNAB= Fine-needle Aspiration Biopsy.

Genetic analysis for LGPA may or may not show a small number of recurring changes involving gene loss in 1p, 6q, 8q, and 13q, and gene gain in 9p regions of the mentioned chromosomes^(7)^. The only recurring change was identified in the copy number in the case of carcinoma ex pleomorphic adenoma (CXPA) in the 22q 12.3-qter gain. In a detailed analysis that was conducted, two primary target genes were identified, nuclear factor I/B (NFIB) and platelet-derived growth factor subunit B (PDGFB), which may be activated as a result of copy number gain involving 9p and 22q chromosomes, respectively^(7)^. Pleomorphic Adenoma Gene 1 (PLAG1) translocation was often over-expressed in LGPA and less often in CXPA^(7,31,38,39,40)^, and high mobility group A2 (HMGA2) was only overexpressed in a small LGPAs subset^(7,31,38,39)^. These and other findings lead to the conclusion that LGPA, salivary gland pleomorphic adenoma, and CXPA have similar genetic and clinical profiles^(7,38,39)^. nase/STAT3 pathway in the normal lacrimal gland, LGPA, and CXPA tissue types. The continuous activity of this pathway results in phosphorylation, and thereby activation of STAT3 as a transcription factor. This leads to the expression of STAT3-regulated genes that are involved in cell growth, survival, and epithelial-mesenchymal transition. The studies showed this pathway to be overexpressed in LGPA and even more so in CXPA, thus indicating the significance of this signaling pathway in the growth of these tumors^(41)^.

Studies by Andreasen et al. described the expression pattern of all components in the interleukin-6/Janus ki-

The carcinoma component might completely cover the pre-existing LGPA, whereas in some cases, only a hyalinized nodule without epithelial elements might be observed. This raises the possibility of a pre-existing LGPA component; however, pathologists are often reluctant to accept enough evidence for a diagnosis of CXPA in these cases. Moreover, CXPA and LGPA share a broad spectrum of histological features (such as nuclear pleomorphism, mitotic activity, and myoepithelial cells with ductal structures), and differentiating them based on these morphological and histopathological features may be difficult. Therefore, the evaluation of genetic alterations by methods, such as fluorescence *in situ* hybridization (FISH), ancillary tests for *PLAG1*, or *HMGA2* gene alterations can be used to distinguish between CXPA and its *de novo* counterparts, as well as separate LGPA from its morphological mimics^(42,43)^.

Zhang et al. reviewed 64 cases of LGPA and 15 of CXPA through immunohistochemical assays. They found that ductal cells in LGPA were positive for pan-cytokeratin and negative for vimentin. The myoepithelial component proved positive for vimentin and negative for pan-cytokeratin. Conversely, in CXPA the myoepithelial component was positive for both pan-cytokeratin and vimentin. Furthermore, the average Ki67 (a nuclear protein associated with cellular proliferation) and C-myc (an oncogene) showed increased expression in CXPA compared to the case of LGPA. As a result, the authors suggested that the immunohistochemical antibodies for C-myc, Ki-67, pan-cytokeratin, and vimentin might provide clues in the differential diagnosis of LGPA and CXPA^(44)^.

The pseudocapsule that surrounds the LGPA is a very fine envelope, approximately tens of micrometers thick, easy to break when manipulated, and it can cause tumor cells to spread over the normal tissues when it breaches^(45)^. LGPA causes smooth and shallow lacrimal fossa bone remodeling^(1,6,13)^. This initial lesion keeps the periosteum intact and covered by the tumor’s pseudocapsule. As a result, during the recurrence of benign nodules of LGPA, they become capable of inducing focal areas of deep erosion or bone remodeling^(13)^. By contrast, the LGPA that develops on the lacrimal gland palpebral lobe does not show bone or ocular globe changes^(12)^. The LGPA lobe orbital, the most common type, generally leads to lacrimal fossa expansion and ocular globe compression^(12)^. Clarós et al. in their study conducted over 15 years, mentioned very few cases with bone erosion (5, 8%), and among them, just one case was reported that showed infiltration of the surrounding tissue^(46)^.

Liu et al. elaborated on the application of Contrastenhanced ultrasound (CEUS) and color Doppler ultrasound in diagnosing lacrimal apparatus tumors. The ultrasound contrast agent intravascularly works and displays the microcirculation within a tumor, making CEUS useful to assess tumor perfusion. This method is better than the color Doppler ultrasound, which lacks reliability since it has low sensitivity to weak blood flows^(5)^. LGPA appears on ultrasound as a round or oval solid mass above the orbit, having a clear edge and dense and uniform echo inside. A small number of LGPA masses may have unclear edges and a nonuniform echo with scattered calcification^(5)^. It is not compressed and has a small number of blood flow signals^(5)^. Rapid filling of contrast within the LPGA mass is noted using CEUS, most showing uniform enhancement, while few of them show concentric uniform or nonuniform enhancement^(5)^. After complete enhancement, the contrast agent slowly fades in LGPA^(5)^. However, adenoid cystic carcinoma of lacrimal gland, the most common malignant lesion of the lacrimal gland, has unclear edges and irregular form on ultrasound. Moreover, in CEUS the contrast agent in the mass rapidly fills and, after its peak, the contrast rapidly gets extinct^(5)^.

**Figure 2:**
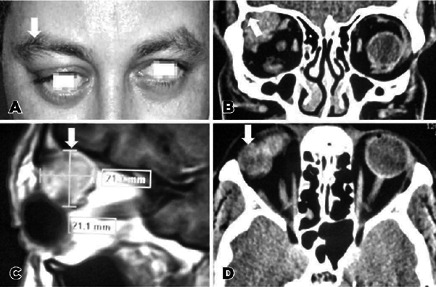
Pleomorphic adenoma of the lacrimal gland. (A) Patient with right-sided proptosis (arrow). (B) Coronal computed tomography (CT) evidencing the erosion of the orbital roof (arrow). (C) Sagittal magnetic resonance imaging (MRI) shows tumor size (arrow). (D) Axial CT presents a large enhancing mass with irregular borders of the right lacrimal gland (arrow).

Though CT technique demonstrates an orbital isodense lesion in the superior lateral aspect of the orbit, magnetic resonance imaging (MRI) is a superior and a more valuable tool in the diagnosis of LGPA (Figure 2). Clarós et al. indicated that among the 52 cases they studied, MRI mostly showed lesions that were isointense to muscle on T1 (96.2%) and hyperintense to muscle on T2-weighted images^(4,47)^ (94.2%). Further, only three cases (5.8%) showed infiltration of periorbital tissue^(46)^.

Diffusion-weighted (DW) MRI has also been described as a useful tool to differentiate benign from malignant lacrimal lesions^(48,49)^. It is based on the molecular water motion of the tissue, which is changed by pathological processes. Apparent diffusion coefficient (ADC) calculated from the DW images can be used to differentiate these lesions. Benign lesions have higher ADC values, owing to their lower cellularity, than malignant lesions^(4,48,49)^. Elkhamary found a mean ADC value of 1.21 ± 0.03 · 10^-3^ mm^2^/s for LGPAs, while the mean ADC value for malignant lacrimal gland lesions was 0.76 ± 0.14 · 10^-3^ mm^2^/s^(48)^. Conversely, Ahmed et al. found an ADC value of 1.8 · 10^-3^ mm^2^/s for LGPA and 1.2 · 10^-3^ mm^2^/s for malignant lacrimal gland tumors^(49)^. Elkhamary established a cut-off level of 0.90 · 10^-3^ mm^2^/s to differentiate benign from malignant lesions, with an accuracy of 90% and an area under the curve of 0.95^(48)^, while Ahmed et al. established the cut-off value at 1.25 · 10^-3^ mm^2^/s^(49)^. Further radiological investigations suggested malignancy, including invasion of the bone cortex^(1,13,48)^, ill-defined tumor extension outside of the lacrimal gland, and molding around the globe^(12)^. Watanabe et al. identified several cases of LGPA depicting rim calcification although this imaging feature was not reported by others^(12)^. The primary clinical findings of LGPA are summarized in table 2.

**Table 2:** Clinical characteristics of LGPA in four series of cases

Clinical features	Watanabe et al.^(12)^ n=36	Clarós et al.^(46)^ n=52	Liu et al.^(5)^ n=109	Ye şiltaş et al.^(52)^ n=14	Total n=211 (%)
Exophtalmos	28	27	71	9	135 (64,0)
Ptosis	30	20		1	51 (24,2)
Diplopia	28	9	4		41 (19,4)
Epiphora		7	9		16 (7,6)
Reduced ocular ductions		7	25		32 (15,2)
Ocular discomfort	2	6	1		9 (4,3)
Decreased visual acuity		4	12		16 (7,6)
Ocular dryness		2			2 (0,9)
Conjunctival hyperaemia		2			2 (0,9)
Periorbital sensory loss		1			1 (0,5)
Raised intraocular pressure		1			1 (0,5)

According to Wiktorin et al., 210 out of 225 fine-needle aspiration biopsy (FNAB) samples analyzed at the Division of Clinical Cytology, Karolinska Hospital, Sweden, between the years 2005 and 2013, showed the presence of orbital lesions. This indicated an 87% success rate of the cytologic diagnosis derived from FNAB as compared to the histopathologic diagnosis from the incisional or excisional biopsy. A total of 43 patients with tumors could be compared using FNAB cytologic diagnosis and the histopathologic biopsy diagnosis. FNAB diagnosis was useful in correctly diagnosing 36 of them; further, 5 cases were inconclusive and only 2 cases were misdiagnosed as normal, and showed inflammation^(50)^.

## DISCUSSION

As suggested by the literature survey, among the treated cases of LGPA, the accurate diagnosis, followed by surgical treatment, and total resection of the tumor-keeping the pseudocapsule intact-is the best therapeutic option. This approach has great prognosis, lower morbidity and mortality, and lower recurrence risk^(1,11,13,17,19,20,22,27,29,38,43,45)^. The total resection occasionally can lead to the removal of the primary lacrimal gland, the main producer of tear fluid. However, there is compelling evidence that the structures within the ocular surface are capable of maintaining adequate tear secretion^(51)^.

Several studies have suggested that performing a biopsy may lead to inadequate management of LGPA and worsening the prognosis^(1,13,17,19,20,22,27,29,38,45)^. The pseudocapsule rupture during surgery (in an attempt to remove the tumor) or biopsy, may cause an increased recurrence risk of this adenoma, and possibly its malignization, typically converting it to CXPA^(3,20,22,27,38,46)^. Yeşiltaş et al. in their retrospective review of 92 patients with lacrimal gland tumors, conducted between the years 1999 and 2017, found 14 LGPAs to be recurrent. Among them, four cases had undergone subtotal excision wherein breach of tumor pseudocapsule was noted during surgery. Three of these LGPAs recurred at a mean of 95.9 months (range 40-185)^(52)^.

Thus, six mentions of previous biopsy^(16,22,23,24,25,26)^ were found among the 24 cases reports (Table 1), and no report of recurrence. Moreover, Wiktorin et al described 43 other FNAB in tumors^(50)^.

FNAB practice is often associated with greater morbidity and mortality, Wiktorin et al. concluded that it is no longer tenable to continue a strict “no biopsy” policy for suspected LGPA, in reference to FNAB (and not incisional biopsy)^(50)^. In summary, the apparent resistance to the use of FNAB in the orbit seems to be related to reports in the 1980s, in which globe perforation with damages to other structures were described. Since then, cytology methods have been refined, with significant improvements in immunocytochemistry and other associated techniques^(6,50,53)^. Evaluation of genetic alterations by FISH ancillary tests for *PLAG1* or *HMGA2* may represent a valuable tool in the diagnosis of challenging cases; however, this deserves further investigation for its wide acceptance^(42,43,54)^.

Several factors, including incomplete resection during the first surgery, intraoperative spillage of tumor cells, or the natural history of the tumor could be attributed to a risk of relapse^(1,3,6,13,19,22,27,45)^. The literature adequately suggests that biopsy is not necessary if appropriate imaging studies are conducted^(20,38,46)^. With this background, MRI is the preferred method for examination of intracranial infiltration^(20,38,46)^. According to Clarós et al., in LGPA the MRI shows lesions isointense to muscle on T1 and hyperintense lesions on T2-weighted images^(4,6,46,47)^.

Moreover, areas of bone erosion in LGPA do not necessarily imply the presence of malignant transformation (e.g., CXPA)^(48)^, but this possibility should always be considered in cases of recurrent LGPA.

Ultrasound and CEUS can be used to determine the mass shape, edge, and dimensions. It may also facilitate the diagnosis of the tumor when identifying its enhancement pattern^(5)^. However, a criterion for distinguishing malignant and benign lesions remains unaddressed. DW MRI has proven to be a useful, reliable, safe, and noninvasive way of differentiating LGPAs from malignant lesions. Although studies established different ADC values for LGPA, both articles indicated that LGPA has a statistically significant higher ADC value when compared to malignant lesions^(48,49)^. Elkhamary acknowledged this difference through his study and associated it with different magnetic fields and technical parameters used in the publications^(48)^.

At present, research in the field of genetics indicates that the molecular changes that occur in LGPA are similar to those found in salivary gland tumor pathogenesis^(3,7,38,41,43)^. This is further corroborated by the study done by Andreasen et al., which suggests that future treatments targeting STAT3 could be promising agents for patients with LGPA and CXPA^(41)^.

In particular, the best form of treatment still is prevention for repeated occurrences of LGPA. Preventing the recurrence should be the aim of the LGPA treatment since the initial appearance of the tumor. A recurrent LGPA becomes difficult to manage, may require multiple operations over a wide anatomic area, and may ultimately require radical surgery in the form of orbital exenteration^(38,18,46)^. Thus, the existence of different surgical modalities for this tumor should be understood and adequately explored to enable complete recovery in the patients^(55,56)^. The knowledge of orbital anatomy, careful selection of an appropriate and less invasive approach (depending on the case), and the use of modern available surgical tools may significantly reduce the morbidity in patients with LGPA^(56)^. The surgeon who performs the first surgery in patients with LGPA has the best chance to curb the disease by complete removal of LGPA.
